# Potential Biomarker Peptides Associated with Acute Alcohol-Induced Reduction of Blood Pressure

**DOI:** 10.1371/journal.pone.0147297

**Published:** 2016-01-27

**Authors:** Ichiro Wakabayashi, Mikio Marumo, Daisuke Nonaka, Tomoko Shimomura, Ryoji Eguchi, Lyang-Ja Lee, Kenji Tanaka, Katsuhiko Hatake

**Affiliations:** 1 Department of Environmental and Preventive Medicine, Hyogo College of Medicine, Nishinomiya, Hyogo, 663–8501, Japan; 2 Membrane Protein and Ligand Analysis Center, Protosera Inc., Amagasaki, Hyogo, 660–0083, Japan; 3 Department of Legal Medicine, Nara Medical University, Kashihara, Nara, 634–8521, Japan; National University of Singapore, SINGAPORE

## Abstract

The purpose of this study was to explore the peptides that are related to acute reduction of blood pressure after alcohol drinking. Venous blood was collected from male healthy volunteers before and after drinking white wine (3 ml/kg weight) containing 13% of ethanol. Peptidome analysis for serum samples was performed using a new target plate, BLOTCHIP^®^. Alcohol caused significant decreases in systolic and diastolic blood pressure levels at 45 min. The peptidome analysis showed that the levels of three peptides of *m/z* 1467, 2380 and 2662 changed significantly after drinking. The *m/z* 1467 and 2662 peptides were identified to be fragments of fibrinogen alpha chain, and the *m/z* 2380 peptide was identified to be a fragment of complement C4. The intensities of the *m/z* 2380 and *m/z* 1467 peptides before drinking were associated with % decreases in systolic and diastolic blood pressure levels at 45 min after drinking compared with the levels before drinking, while there were no significant correlations between the intensity of the *m/z* 2662 peptide and % decreases in systolic and diastolic blood pressure levels after drinking. The *m/z* 1467 and 2380 peptides are suggested to be markers for acute reduction of blood pressure after drinking alcohol.

## Introduction

Habitual alcohol drinking is known to show both beneficial and harmful effects on the risk of cardiovascular disease [1,2]. The beneficial effect is mainly explained by alcohol-induced increase in blood HDL cholesterol [3,4]. In addition, attenuation of blood coagulability due to inhibition of platelet aggregation [5] and decrease in fibrinogen levels [6] are also involved in the lower risk of cardiovascular disease in light-to-moderate drinkers than in nondrinkers. On the other hand, alcohol is known to cause hypertension [7,8], which is a major risk factor for cardiovascular disease [9]. Although the exact mechanism for alcohol-induced hypertension remains to be clarified, the most likely hypothesis for the mechanism in heavy drinkers at present is increased sympathetic activity following withdrawal of alcohol [10–12]. In this hypothesis, a single intake of alcohol lowers blood pressure [13,14], and habitual daily alcohol intake induces repeated intermittent alcohol withdrawal, which causes hypertension through sympathetic activation. Although circulating vasodilators such as nitric oxide [15], prostacyclin [16] and kinin [17], as well as acetoaldehyde [18], have been proposed to be involved in alcohol-induced acute vasodilation, the exact mechanism for the hypotensive effect of alcohol also remains to be clarified.

The purpose of this study was to identify circulating peptides that are related to alcohol-induced acute changes in blood pressure. Serum peptides were analyzed by matrix-assisted laser desorption/ionization-time of flight mass spectrometry (MALDI-TOF-MS) using a new target plate (BLOTCHIP^®^), which enables one-step direct electric transfer of analytes from the one-dimensional PAGE (polyacrylamide gel electrophoresis) gel to the target plate [19]. A great merit of this new method is that no pretreatment of blood samples is required, making it possible to avoid removal of large amounts of blood proteins including albumin before analysis and thus resulting in more efficient detection of peptides. We first identified the peptides of which the serum levels changed in relation to alcohol-induced changes in blood pressure. Then we examined whether the blood levels of these peptides before drinking were associated with alcohol-induced acute reduction of blood pressure.

## Subjects and Methods

### Subjects

Subjects were healthy male volunteers aged from 27 to 45 years. The purpose of the study and the protocol of the experiment for drinking alcohol were explained to all of the participants. Written informed consent was obtained from all subjects. The protocol of this study was approved by the Hyogo College of Medicine Ethics Committee (No. 1413 in 2015). All of the subjects were nonsmokers.

### Evaluation of individual alcohol sensitivity of subjects

Individual sensitivity to alcohol was surveyed by using a self-administered questionnaire called alcohol sensitivity screening test (ALST) [20]. The scoring system for determination of alcohol sensitivity was originally prepared on the basis of results of a stepwise logistic regression analysis to discriminate between the typical homozygote of aldehyde dehydrogenase 2 (ALDH2*1*1) and its atypical heterozygote (ALDH2*1*2). The questionnaire included three items on symptoms (facial flushing, skin flushing other than facial flushing, and palpitation) that appear when drinking alcohol, and the score of each item was determined by the frequency of each symptom as follows: facial flushing: 3.8 (always occurs), 1.1 (sometimes occurs) or 0 (never occurs); flushing elsewhere: 1.6 (always occurs), 1.1 (sometimes occurs) or 0 (never occurs); palpitation: 2.3 (always occurs), 1.3 (sometimes occurs) or 0 (never occurs). The total score was calculated as the score for the ALST, and the subjects were classified as those with low sensitivity and those with high sensitivity when the ALST score was ≤ 3.1 and > 3.1, respectively.

### Administration of alcohol

On the day of the experiment, each subject drank 3 ml per kg body weight of white wine, containing 13% of ethanol, within 10 min. They had not consumed any alcohol beverages at least for 24 hours before the experiment.

### Measurement of blood pressure and collection of blood samples

Blood pressure was measured at the right brachial artery by using a blood pressure monitor (TERUMO DIGITAL BLOOD PRESSURE MONITOR ES-H55, Terumo, Tokyo, Japan) every 15 min after drinking alcohol. Blood was collected from the left antecubital vein just before drinking and at 45 min and 2–3 hr after drinking. At 45 min after drinking, all of the subjects showed lower systolic and diastolic blood pressure levels than those before drinking. In some subjects, systolic and diastolic blood pressure levels were further lowered from 2 to 3 hr after drinking compared with those at 45 min, and blood was collected from those subjects at 3 hr after drinking. In the other subjects, systolic and/or diastolic blood pressure was increased at 2–3 hr after drinking compared with corresponding blood pressure at 45 min after drinking, and blood was collected from those subjects at the time when the increase(s) of systolic and/or diastolic blood pressure levels compared with the levels at 45 min after drinking was detected initially from 2 to 3 hr after drinking. Thus, blood samples were collected during the period from 2 to 3 hr after drinking depending on the changes in blood pressure of each subject after drinking. Blood samples collected were immediately transferred to plastic tubes with and without 3.2% sodium citrate at a volume ratio of 1 (sodium citrate solution) to 9 (blood). After centrifugation at 1500 x g for 10 minutes, each serum or plasma sample obtained was stored in a freezer at -80 degrees Celsius until analysis.

### Peptidome analysis

Serum peptidomic analysis was conducted by using a newly-established one-step direct transfer technology, “BLOTCHIP^®^-MS analysis”, as described elsewhere [19,21,22]. Briefly, peptides and proteins in serum samples were separated using sodium dodecyl sulfate (SDS)-polyacrylamide gel electrophoresis and then electroblotted onto BLOTCHIP^®^ (Protosera Inc., Amagasaki, Japan). MALDI matrix, α-cyano-4-hydroxycinnamic acid (CHCA) (Sigma-Aldrich Co., MO, USA), was applied directly onto BLOTCHIP^®^ and peptidome profiles were obtained in a linear mode of UltraFlexII TOF/TOF (Bruker Daltonics Inc. MA, USA). All sample measurements were repeated four times. Statistical analyses of MS spectral data were conducted using ClinProTools version 2.2 (Bruker Daltonics). Significantly different peaks among all possible pairs of the subject groups (before drinking, 45 min after drinking, 2–3 hr after drinking) were detected by the Wilcoxon signed-rank test, a nonparametric test for 2-group comparisons within the software. Structural analysis of the significantly different peaks was conducted as previously described [21]. For peptide identification, MASCOT software version 2.1 was used for a "MS/MS ions search". Parent peptide and MS/MS ions tolerance parameters were set at ±100 ppm and ±0.7 Da, respectively. The SwissProt sequence database, of which the taxonomy was limited to “humans”, was selected for the searches. The proteolytic enzyme parameter was set to “None”. “Oxidation” and “phosphorylation” were selected as variable modifications. The peptide identification criteria for this work were based on a probability-based MOWSE scoring algorithm and the significant threshold was set to *p* < 0.05.

### Measurement of blood ethanol

Serum ethanol levels were measured by gas chromatography by using Gas chromatograph GC-17A (Shimadzu, Kyoto, Japan).

### Measurement of complement C4 and complement C4a anaphylatoxin

Serum complement C4 levels were measured by a turbidimetric immunoassay using a commercial kit (N-assay TIA C4-SH Nittobo, Nittobo, Tokyo, Japan). Complement C4a anaphylatoxin concentration in plasma was measured by an enzyme immunoassay using a commercial kit (Human C4a ELISA Kit, BD Biosciences, New Jersey, USA).

### Statistical analysis

Statistical analyses were performed using a computer software program (SPSS version 16.0 J for Windows, Chicago IL, USA). Blood pressure and intensity of each peptide were analyzed by using repeated one-way analysis of variance and Friedman’s test, respectively, and were compared at two different time points by using the paired Student’s t test and Wilcoxon signed-rank test, respectively. In linear regression analysis, Spearman’s rank correlation coefficients (*r*) were calculated. A probability of *p* < 0.05 was considered statistically significant.

## Results

### Characteristics of subjects

Table 1 shows the characteristics of the subjects. No abstainers were included in the subjects. Seven subjects and three subjects were regular and occasional drinkers, respectively. Among the seven regular drinkers, four and three were estimated to have typical homozygote (active type) and atypical heterozygote (inactive type) of ALDH2, respectively, according to the results of ALST. Among the three occasional drinker subjects, two subjects and one subject were estimated to have active and inactive types of ALDH2, respectively.

**Table 1:** Characteristics of subjects.

Subject	Age	Weight	Amount of wine	Systolic BP	Diastolic BP	Score of ALST	Drinker
(No.)	(years)	(kg)	(ml)	(mmHg)	(mmHg)	(estimated ALDH2*)	
1	38	66	198	120	73	0 (active)	Regular
2	45	69	207	149	91	3.5 (inactive)	Regular
3	38	65	195	135	88	2.2 (active)	Regular
4	38	70	210	117	67	7.7 (inactive)	Regular
5	37	80	240	121	75	6.2 (inactive)	Regular
6	37	66	198	113	72	1.1 (active)	Occasional
7	32	78	234	141	79	5.4 (inactive)	Occasional
8	27	67	201	130	81	0 (active)	Occasional
9	33	83	249	118	76	0 (active)	Regular
10	33	63	189	125	76	1.1 (active)	Regular
Overall	35.8 ± 4.8	70.7 ± 7.0	212.1 ± 21.1	126.9 ± 11.6	77.8 ± 7.3	1.65 (0–7.7)	—

BP, blood pressure; ALST, alcohol sensitivity screening test; ALDH, aldehyde dehydrogenase. Activity of ALDH2 was estimated by using the ALST: active, ALST score ≤ 3.1; inactive, ALST score > 3.1. Regular drinkers and occasional drinkers were defined as those drinking alcohol once or more and less than once a week, respectively. Mean ± standard deviation or median with range indicated in the parenthesis of each variable is shown for overall subjects.

### Changes in blood pressure after drinking alcohol

**Fig 1:**
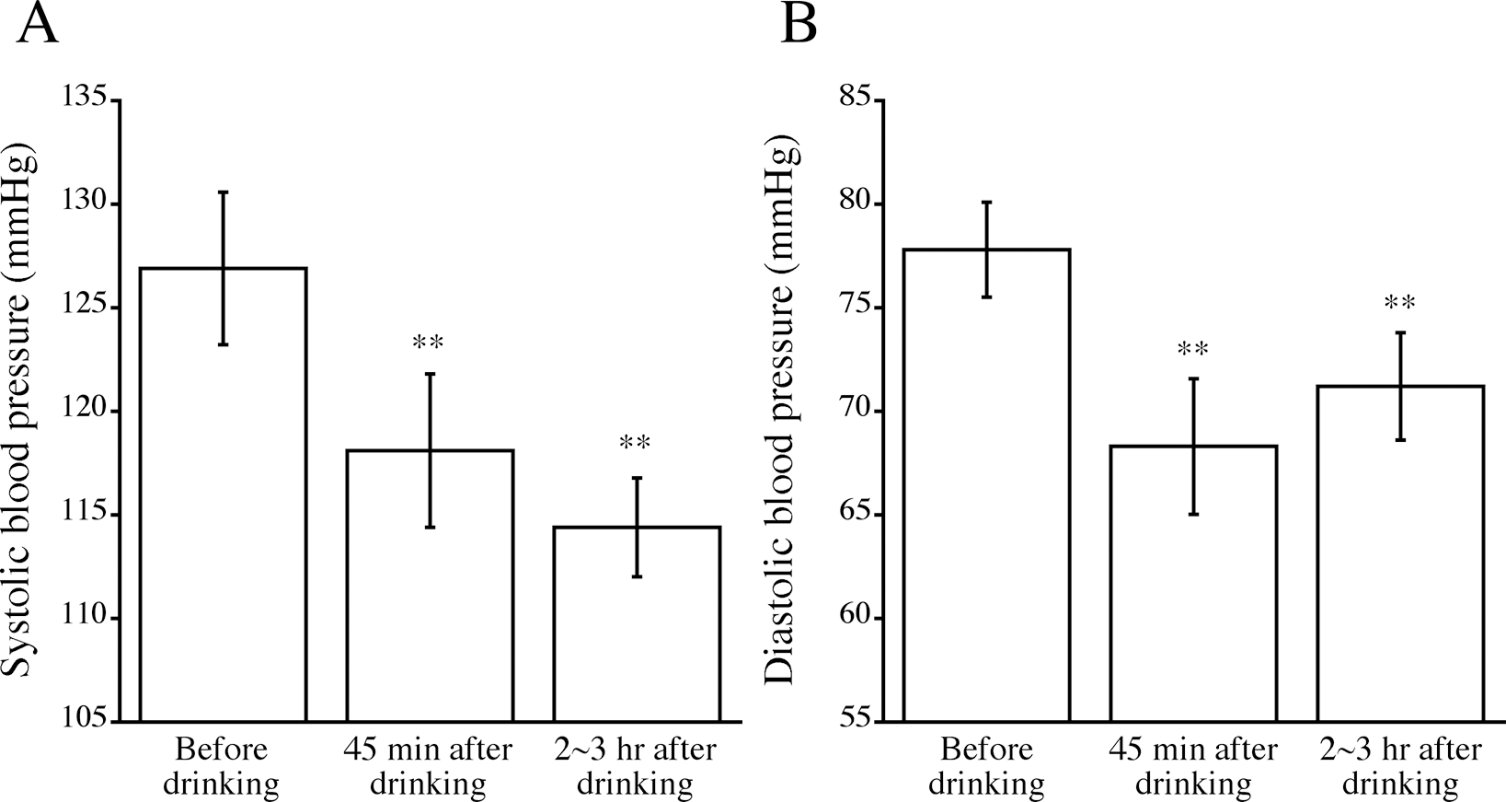
Changes in systolic (A) and diastolic (B) blood pressure levels after drinking alcohol. Blood pressure was measured just before dinking and at 45 min and 2–3 hr after drinking. Means ± standard errors of blood pressure levels are shown. Asterisks denote significant differences from the levels before drinking (**, *p* < 0.01).

Fig 1 shows changes in blood pressure after alcohol drinking. Systolic and diastolic blood pressure levels were significantly lower at 45 min and 2–3 hr after drinking than the levels before drinking. There were no significant differences in systolic and diastolic blood pressure levels at 45 min and at 2–3 hr after drinking.

### Relationships between blood ethanol concentration before drinking and acute changes in blood pressure after drinking

The mean ethanol concentration at 45 min after drinking was 0.29 ± 0.09 mg/ml. Blood ethanol level showed a significant correlation with % change in systolic blood pressure at 45 min after drinking (Spearman’s rank correlation coefficient, -0.69 [*p* = 0.026]), but the correlation of blood ethanol level with % decrease in diastolic blood pressure at 45 min after drinking was not significant (Spearman’s rank correlation coefficient, -0.30 [*p* = 0.397]).

### Detection of peptides in serum that changed in relation to alcohol drinking

**Fig 2:**
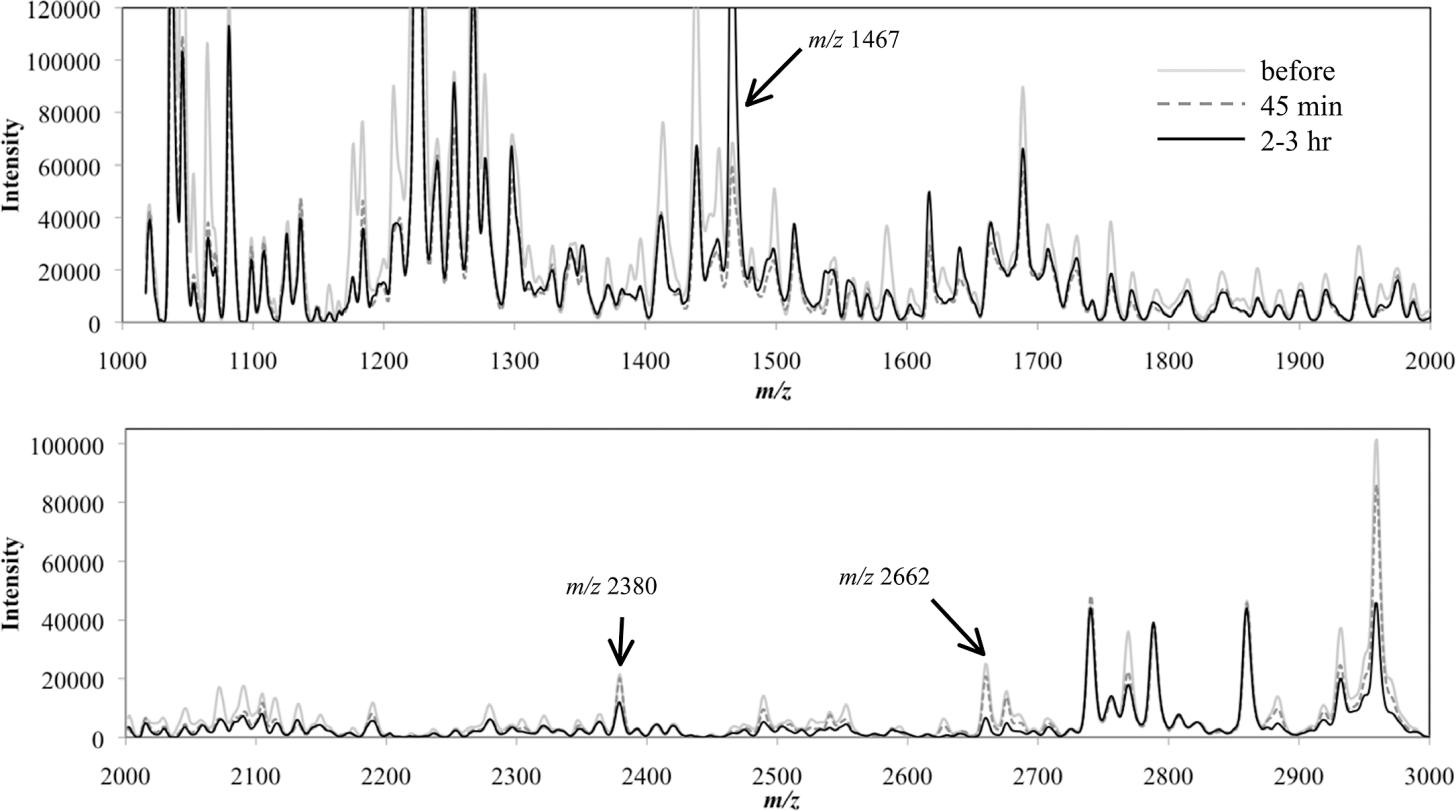
Differential profiling of serum sampled from subjects before and after drinking. Each integrated spectrum normalized with ClinPro Tools version 2.2 is the average of 10 samples (total of 44 integrations) from subjects.

**Fig 3:**
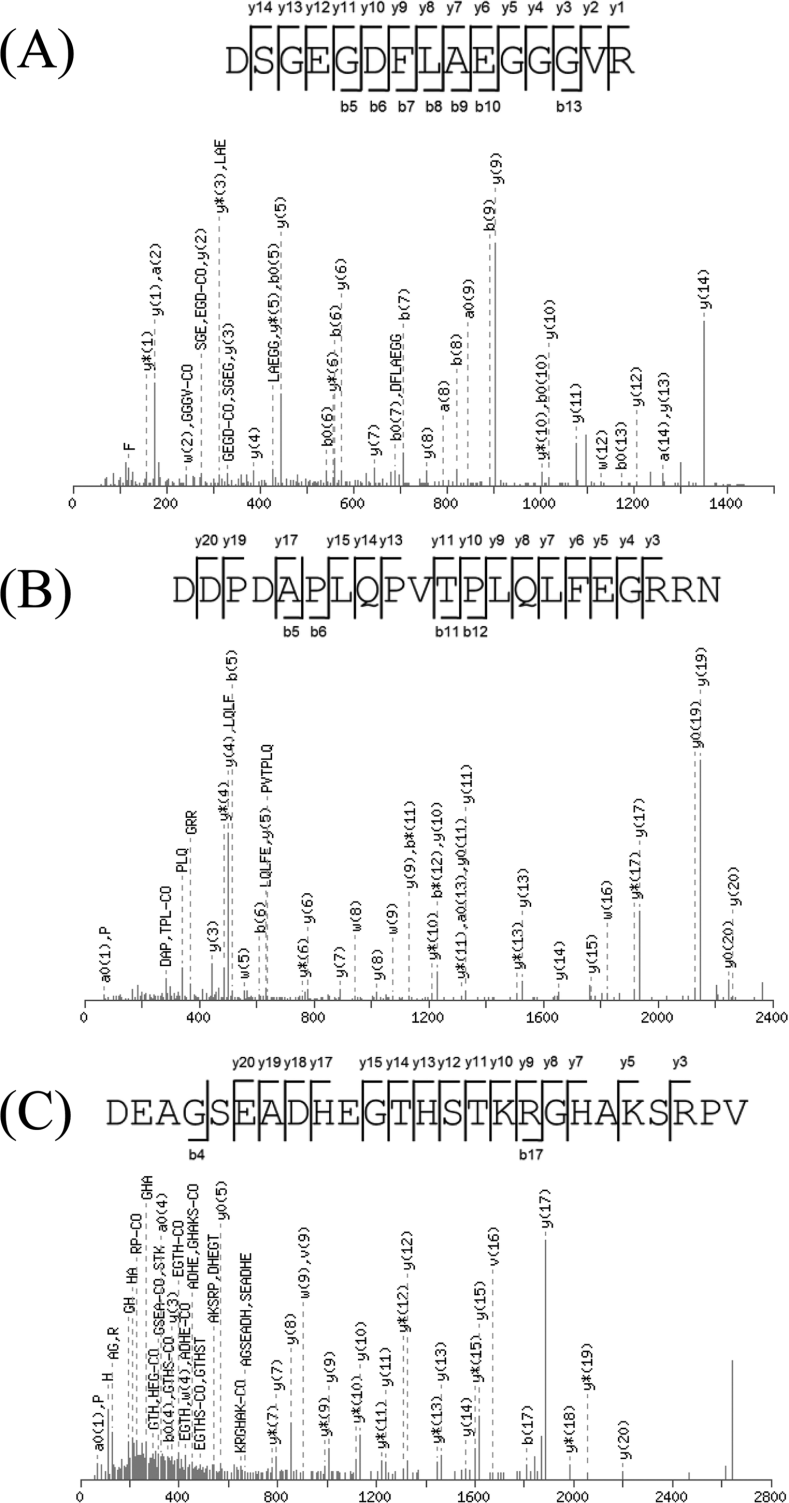
MS/MS structural analysis of *m/z* 1467 (A), 2380 (B), 2662 (C) peptides in the sample after reversed-phase high-pressure liquid chromatography separation.

Fig 2 shows a superimposition of average MS spectra of each group (before drinking, 45 min after drinking, or 2–3 hr after drinking) obtained by MALDI-TOF-MS analysis of the serum peptides transferred to BLOTCHIP^®^. By differential analysis among the three groups using ClinProTools 2.2 software, we found that levels of eighteen peptides were statistically different in the groups of “45 min after drinking” and “2–3 hr after drinking” compared with the levels in the group before drinking. To obtain more information about the peptides, we conducted off-line liquid chromatography-MS/MS analysis of the serum peptides. Observed *m/z* values of the peptides were widely spread over the range between 1k Da and 13k Da. We tried to purify all of the peptides, but only three (*m/z* 1467, 2380 and 2662) were successfully purified by reversed-phase chromatography and a sufficient amount of the peptides was obtained to carry out MALDI-TOF-MS/MS analysis. MALDI-MS/MS structural analysis of each peptide exhibited consecutive fragment ion series derived from their own sequence (Fig 3). A MASCOT search revealed that *m/z* 1467 and 2662 peptides were fibrinogen alpha chain fragments derived from the N-terminal and C-terminal regions of the protein, respectively, and that the *m/z* 2380 peptide was a fragment of complement C4 (Table 2). For further evaluation of statistical significance of the identified peptides, we analyzed FDR of the overall comparison using the Twilight package within R statistical software [23]. At FDR < 0.1, 9 peaks out of the 18 peaks remained. At FDR < 0.05, 6 peaks still remained. In both cases, two of three identified peptides, peptides *m/z* 2380 and 2660, were still acceptable (*p* = 0.00151 and 0.0000433, respectively, between “before drinking” and “at 2–3 hr after drinking”) even under FDR control. Although peptide *m/z* 1467 was significant (*p* = 0.0288) only at FDR < 0.25, we accepted this peptide as the subject of the study because of its previous appearance as an important peptide in a study related to alcohol intake [24].

**Table 2:** Profiles of the identified peptide fragments showing quantative changes in relation to changes in blood pressure after alcohol drinking.

Monoisotopic	Mascot MOWSE	Origin of the peptide	Amino acid number	Peptide sequence
[M+H]^+^	score		(N-/C-terminus)	
1465.626	115	Fibrinogen alpha chain	21–35	DSGEGDFLAEGGGVR
2378.134	124	Complement C4-A	1429–1449	DDPDAPLQPVTPLQLFEGRRN
2659.161	82	Fibrinogen alpha chain	605–629	DEAGSEADHEGTHSTKRGHAKSRPV

### Changes in intensities of the peptides with *m/z* 1467, 2380 and 2662 after drinking alcohol

**Fig 4:**
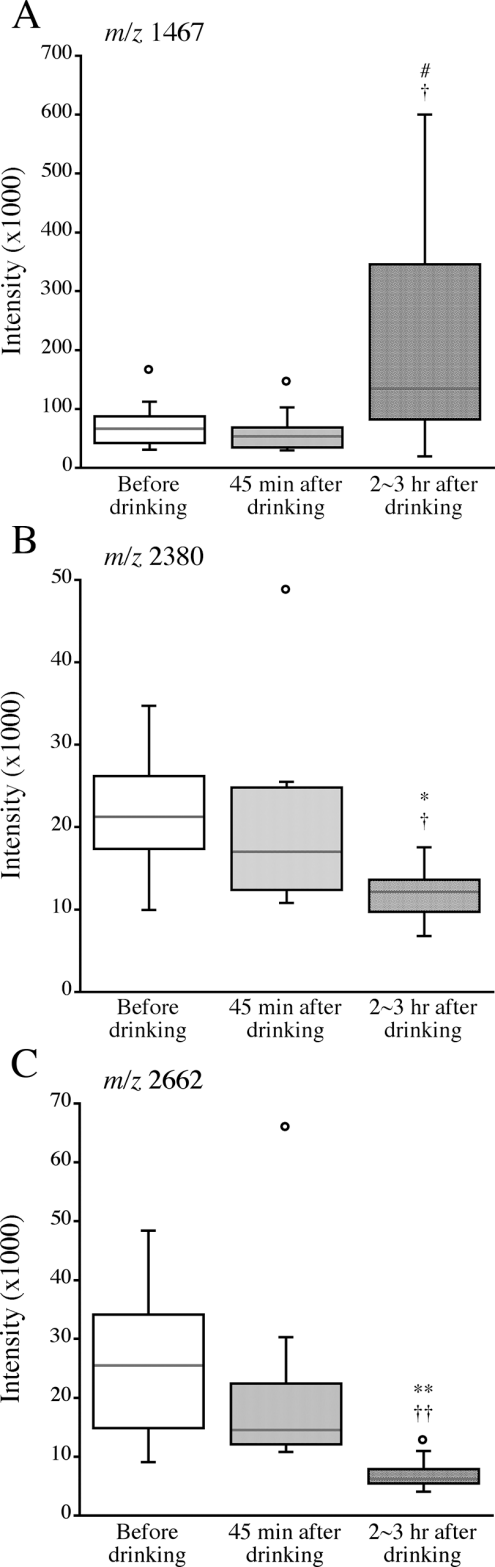
Changes in intensities of *m/z* 1467 (A), 2380 (B) and 2662 (C) peptides after drinking alcohol. Medians with 25 and 75 percentile values of intensities of the peptides are shown using box plots. Intensities of each peptide in serum collected just before dinking and at 45 min and 2–3 hr after drinking were measured by mass spectrometry using BLOTCHIP^®^. Symbols denote significant differences from the levels before drinking (*, *p* < 0.05; **, *p* < 0.01) and the levels at 45 min after drinking (†, *p* < 0.05; ††, *p* < 0.01). #, a marginally significant difference from the intensity before drinking (*p* = 0.074).

As shown in Fig 4, the intensities of *m/z* 2380 and 2662 peptides were significantly lower at 2–3 hr after drinking than the corresponding intensities before drinking and those at 45 min after drinking. The intensity of the *m/z* 1467 peptide was significantly higher at 2–3 hr after drinking than the intensity at 45 min after drinking, and there was a marginally significant difference (*p* = 0.074) between the intensity of the *m/z* 1467 peptide before drinking and that at 2–3 hr after drinking.

### Relationships between each peptide level before drinking and acute changes in blood pressure after drinking

**Fig 5:**
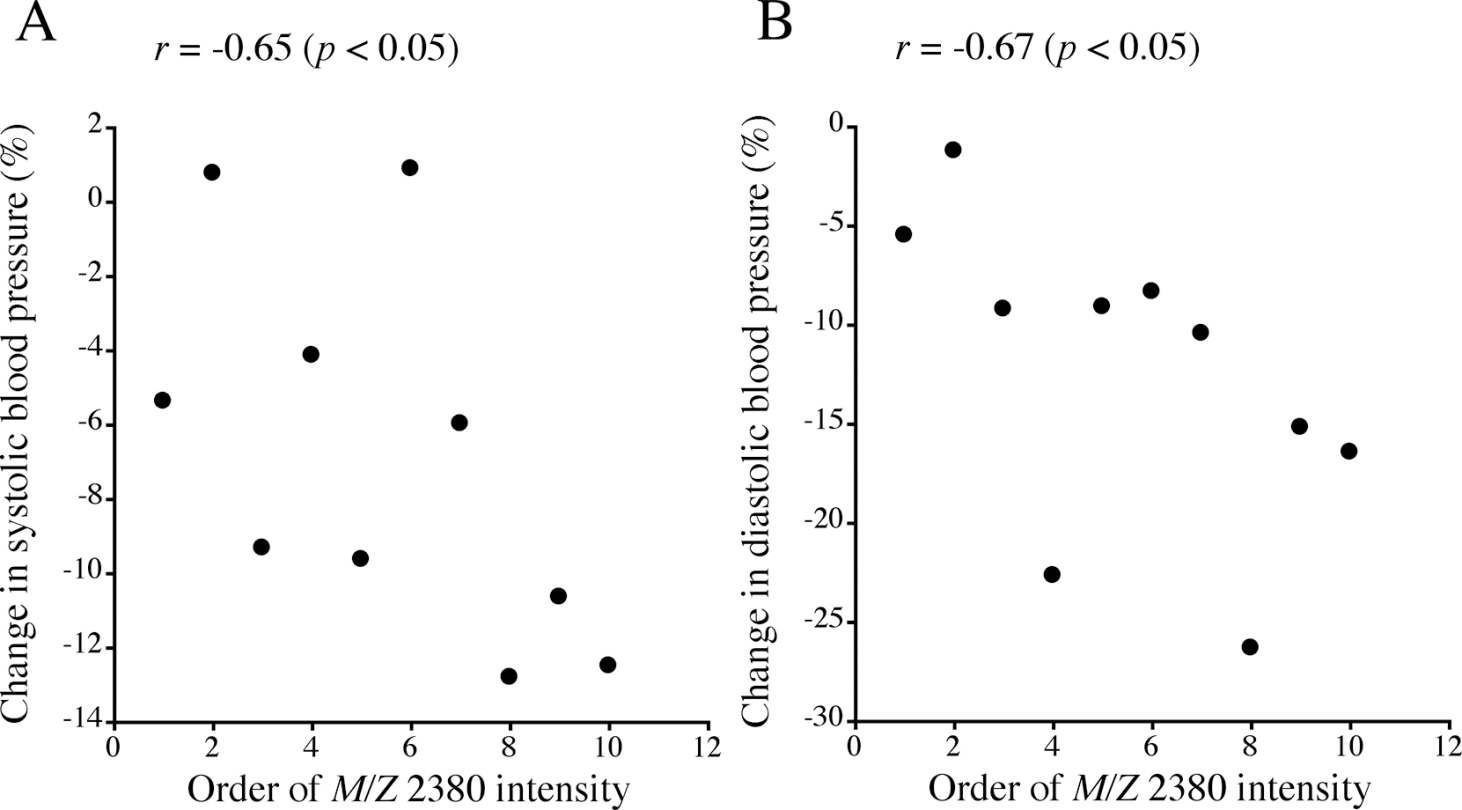
Scatter plots of the relationships between ranks of serum *m/z* 2380 peptide intensity before drinking and % changes in systolic (A) and diastolic (B) blood pressure at 45 min after drinking. Spearman’s rank correlation coefficients (*r*) are given in the figures.

The intensity of the *m/z* 2380 peptide in blood before drinking showed a significant inverse correlation with % changes in systolic and diastolic blood pressure levels at 45 min after drinking (Table 3, Fig 5). The intensity of the *m/z* 1467 peptide showed a significant correlation with % change in systolic blood pressure levels and a marginally significant correlation with % change in diastolic blood pressure levels (Table 3). However, there were no significant correlations of the intensity of the *m/z* 2662 peptide with % changes in systolic and diastolic blood pressure levels (Table 3).

**Table 3:** Correlations between each peptide level in blood before drinking and changes in systolic and diastolic blood pressure levels before and after drinking.

	% change in systolic BP	% change in diastolic BP
*m/z* 1467	-0.65 (*p* = 0.043)	-0.59 (*p* = 0.074)
*m/z* 2380	-0.65 (*p* = 0.043)	-0.67 (*p* = 0.033)
*m/z* 2662	-0.50 (*p* = 0.138)	-0.48 (*p* = 0.162)

Spearman’s rank correlation coefficients with their *p* values in the parentheses are shown. Variables for analysis were each peptide level in blood before drinking and % changes in blood pressure levels at 45 min after drinking compared with those before drinking. BP, blood pressure.

### Relationships of serum complement C4 level with intensity of the *m/z* 2380 peptide and acute changes in blood pressure after drinking

The mean with its standard deviation of serum complement C4 levels in the subjects before drinking was 27.8 ± 6.0 mg/dl (range: 18–38 mg/dl). The intensity of the *m/z* 2380 peptide, a fragment of complement C4, before drinking showed a marginally significant inverse correlation with serum C4 level before drinking (*r* = -0.62 [*p* = 0.056], Fig A in S1 Fig). There were no significant correlations of serum C4 level before drinking with % changes in systolic and diastolic blood pressure levels at 45 min after drinking (Pearson’s correlation coefficient: systolic blood pressure, *r* = 0.45 [*p* = 0.190], Fig B in S1 Fig; diastolic blood pressure, *r* = 0.28 [*p* = 0.440], Fig C in S1 Fig).

### Relationships between complement C4a anaphylatoxin level in plasma before drinking and acute changes in blood pressure after drinking

**Fig 6:**
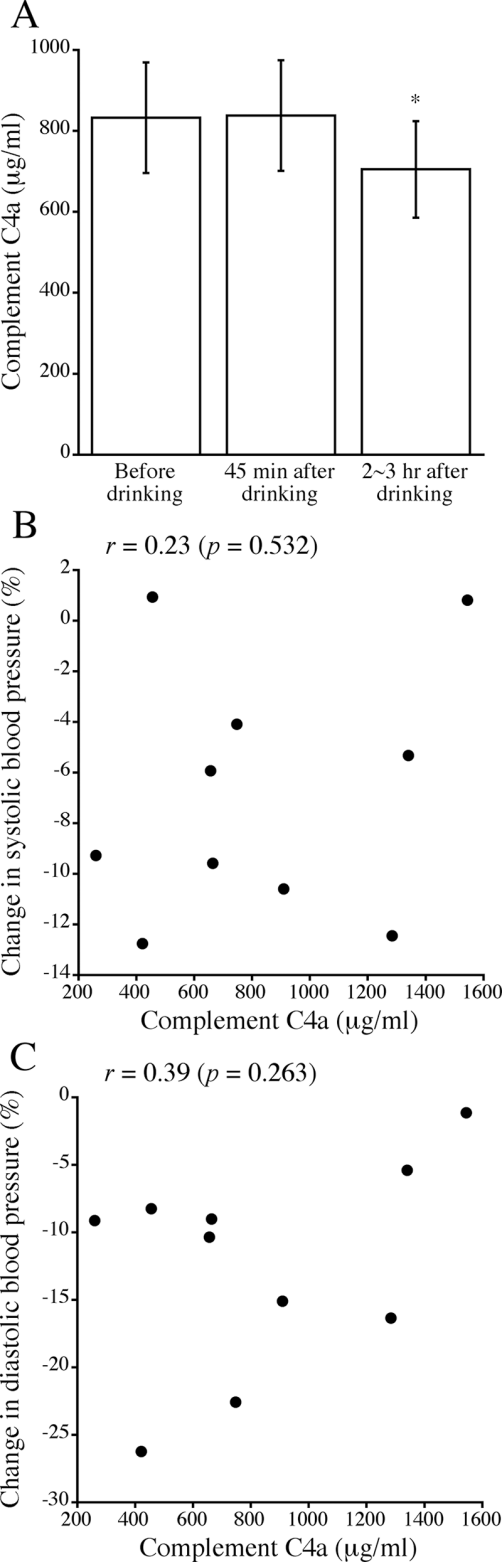
A. Changes in blood complement C4a level after drinking alcohol. Complement C4a concentration was measured just before drinking and at 45 min and 2–3 hr after drinking. Means ± standard errors of complement C4a levels are shown. An asterisk denotes a significant difference from the level before drinking (*, *p* < 0.05). B,C. Scatter plots of the relationships between blood complement C4a level before drinking and % changes in systolic (B) and diastolic (C) blood pressure at 45 min after drinking. Pearson’s correlation coefficients (r) are given in the figures.

Fig 6A shows comparison of serum complement C4a anaphylatoxin levels before and after drinking. The complement C4a level at 2–3 hr after drinking was significantly lower than that before drinking, while there was no significant difference in the complement C4a levels before and at 45 min after drinking (Fig 6A). Thus, the trend of change in complement C4a levels after drinking was similar to the trend of change in the *m/z* 2380 peptide (Fig 4B). As shown in Fig 6B and 6C, there were no significant correlations between complement C4a level before drinking and % changes in systolic and diastolic blood pressure levels at 45 min after drinking (Pearson’s correlation coefficient: systolic blood pressure, *r* = 0.23 [*p* = 0.532]; diastolic blood pressure, *r* = 0.39 [*p* = 0.263]).

### Relationships of ALST score with changes in blood pressure after drinking

The score of ALST was not significantly correlated with % changes in systolic and diastolic blood pressure levels (Spearman’s rank correlation coefficient: systolic blood pressure, *r* = 0.10 [*p* = 0.787]; diastolic blood pressure, *r* = 0.23 [*p* = 0.515]).

## Discussion

By a new peptidomic analysis using BLOTCHIP^®^, a recently developed target plate [19], three peptides in blood, which changed in relation to alcohol-induced acute reduction of blood pressure, were identified to be fibrinogen alpha chain and complement C4 by offline LC-MALDI MS/MS. Serum levels of *m/z* 1467 and 2380 peptides (fragments of fibrinogen and complement C4, respectively) before drinking were significantly correlated with acute reduction of blood pressure after drinking. Thus, *m/z* 1467 and 2380 peptides are proposed to be markers for alcohol-induced reduction of blood pressure. This is the first study in which peptides related to alcohol-induced change in blood pressure were identified. Blood levels of peptides are thought to be determined by activities of peptidases and/or their inhibitors [25]. Therefore, alcohol is speculated to modulate the balance between peptidase and peptidase inhibitor activities in blood.

Although the exact mechanism for hypertension due to habitual alcohol consumption remains to be clarified, the most likely hypothesis for hypertension in heavy drinkers at present is repeated sympathetic stimulation following alcohol withdrawal [10–12]. Therefore, alcohol-induced reduction of blood pressure as an acute effect of alcohol is speculated to be also associated with alcohol-induced elevation of blood pressure as a chronic effect of alcohol in heavy drinkers. Thus, *m/z* 1467 and 2380 peptides are also possible markers for alcohol-induced hypertension. In order to test this hypothesis, further studies investigating the relationships between peptide levels and hypertension by using a conventional method for measuring these peptides are needed in the future.

By the peptidome analysis, change in the level of another peptide, *m/z* 2662 peptide, as well as *m/z* 1467 and 2380 peptides, in relation to blood pressure after drinking was detected. However, no significant correlations were obtained between the *m/z* 2662 peptide level before drinking and % reduction of systolic and diastolic blood pressure levels after drinking (Table 3). Because of the small number of subjects in this study, further studies are needed to determine whether *m/z* 2662 peptide levels are associated with alcohol-induced blood pressure reduction.

Both *m/z* 1467 and 2380 peptide levels before drinking showed associations with changes in blood pressure after drinking. However, the directions of changes in the *m/z* 1467 and 2380 peptides after drinking were different: the levels of the *m/z* 1467 and 2380 peptides were increased and decreased, respectively, at 2–3 hr after drinking compared with the levels before drinking. Although the reason for the above dissociation of changes in levels of the two peptides after drinking is unknown, one possible explanation is different associations of these peptides with circulatory function after drinking, such as continuation of or reversal from blood pressure reduction. It remains to be clarified whether these peptides, fragments of complement C4 and fibrinogen, have some physiological significance.

The *m/z* 1467 and 2662 peptides, different fragments of fibrinogen alpha chain, which were increased and decreased, respectively, after drinking in the present study, are identical to the peptides in serum from chronic alcoholic patients: *m/z* 1467 and 2662 peptides have been reported to be decreased and increased, respectively, after cessation of drinking for three months in alcoholics [24]. Thus, the directions of changes in these two peptides were opposite in the above study and the present study (Fig 4), which is reasonable because the alcohol drinking behaviors were also opposite in the above study (alcohol cessation) and the present study (alcohol administration). Blood pressure is known to increase transiently after cessation of drinking in alcoholic patients [10,11]. Thus, there is a possibility that the changes in these peptides were also related to changes in blood pressure after alcohol cessation, although no information on blood pressure of subjects was reported by Sogawa et al. [24].

Serum *m/z* 2380 peptide levels were associated with alcohol-induced acute reduction of both systolic and diastolic blood pressure levels. This peptide originates from complement C4, and there is a marginally significant inverse correlation between serum levels of the *m/z* 2380 peptide and complement C4. This may be reasonable since the *m/z* 2380 peptide was thought to be released from complement C4 after drinking, resulting in a decrease of the full-length complement C4. However, no significant correlations were found between complement C4 levels and % changes in systolic and diastolic blood pressure levels. Therefore, serum complement C4, instead of the *m/z* 2380 peptide, could not be a marker for changes in blood pressure after drinking.

Bioactive fragments of complement, such as C3a, C4a and C5a, are released during the course of complement activation and are called anaphylatoxins, which cause anaphylactic shock when produced in large amounts and increase vascular permeability. We therefore investigated the relationships between complement C4a level and blood pressure change after drinking. Interestingly, the trend of change in complement C4a level after drinking was similar to the trend of change in the *m/z* 2380 peptide. However, no significant correlation was found between complement C4a level and alcohol-induced change in blood pressure. Therefore, complement C4a, a complement C4 fragment that is different from the *m/z* 2380 peptide, is thought not to be useful as a biomarker for alcohol-induced blood pressure reduction. It would also be of interest to investigate ethanol-induced protease activity changes and their relations to ethanol-induced blood pressure changes in future.

Araki et al. recently identified seven characteristic peptides in blood from patients with pregnancy-induced hypertension [21]. Among the seven peptides, one peptide was a fragment of complement C4, the levels of which were higher in patients with pregnancy-induced hypertension than in control subjects with normal blood pressure levels, and it is identical to the *m/z* 2380 peptide, which was decreased after alcohol drinking and was associated with alcohol-induced acute reduction of blood pressure in the present study (Fig 4, Table 3). Thus, the *m/z* 2380 peptide seems to be closely related to blood pressure, and it is of interest to investigate the pathophysiological significance of this peptide in other blood pressure-related diseases including cardiovascular events. The same peptides as the *m/z* 2380 peptide in blood and cerebrospinal fluid have been reported to be associated with hepatocellular carcinoma [26] and Alzheimer’s disease [27], respectively. However, the reasons for the associations of these diseases with the fragment peptide of complement C4 remain unknown. Thus, further studies are needed to elucidate how specific or not the peptide biomarkers detected in the present study are for the alcohol-induced blood pressure reduction.

The ALST is known as a useful method for evaluating individual sensitivity to alcohol [20], which is mainly determined by polymorphism of ALDH2 in Asians [28,29], and consists of simple questionnaires on alcohol drinking-induced acute symptoms including skin flushing due to vasodilation [30]. However, scores of ALST were not significantly correlated with changes in blood pressure after drinking in the present study. One possible explanation for this finding is that individual sensitivity to alcohol affects the tone of small arteries or capillaries reflected by skin flushing but not the tone of large arteries reflected by blood pressure. Although the number of subjects in the present study was too small to prove the above hypothesis, *m/z* 1467 and 2380 peptide levels might be a better marker for predicting alcohol-induced reduction of blood pressure than ALST.

In conclusion, changes in three peptides, which are fragments of fibrinogen alpha and complement C4, were demonstrated to be associated with acute reduction of blood pressure after drinking alcohol. Levels of *m/z* 1467 and 2380 peptides before drinking were significantly associated with alcohol-induced reduction of blood pressure and are proposed to be markers for acute circulatory action of alcohol.

## Supporting Information

S1 Fig A. Scatter plots of the relationship between ranks of *m/z* 2380 peptide intensity and complement C4 level before drinking. A Spearman’s rank correlation coefficient (r) is given in the figure. B,C. Scatter plots of the relationships between blood complement C4 level before drinking and % changes in systolic (B) and diastolic (C) blood pressure at 45 min after drinking. Pearson’s correlation coefficients (r) are given in the figures.
(TIF)
