# Supplementary figures and images for: Potential Biomarker Peptides Associated with Acute Alcohol-Induced Reduction of Blood Pressure
Source: PLoS One. 2016 Jan 27;11(1):e0147297. doi: 10.1371/journal.pone.0147297 (PMC4729683; doi:10.1371/journal.pone.0147297)

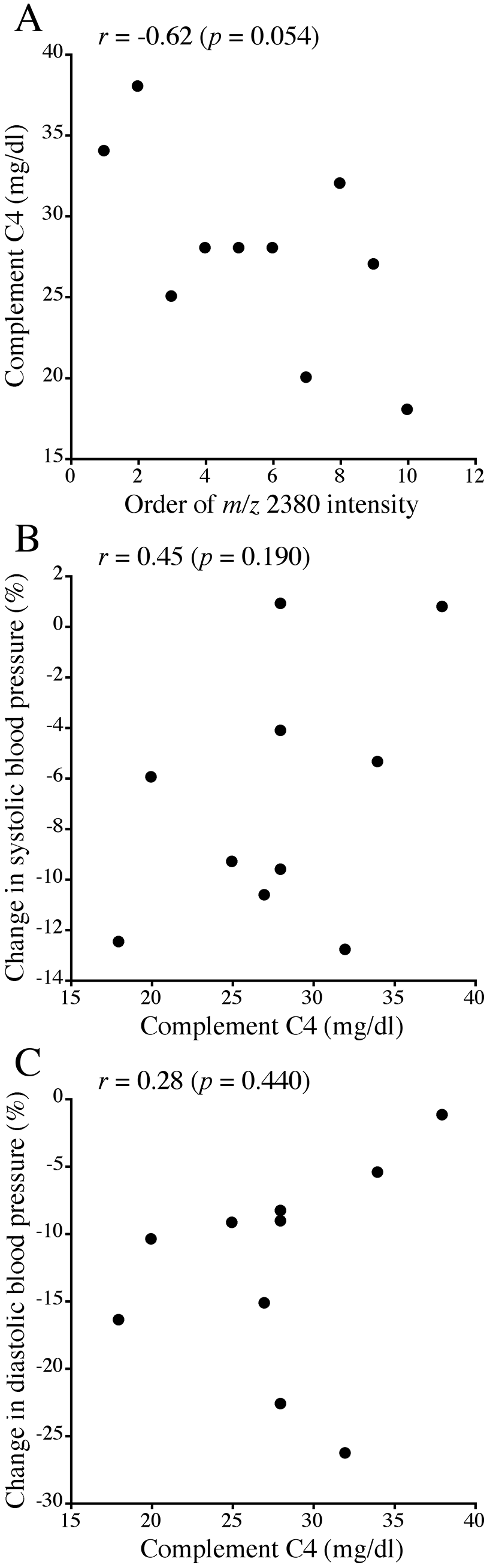

Supplement: S1 Fig — Pearson’s correlation coefficients (r) are given in the figures. (TIF) [file pone.0147297.s001.tif]
